# Long COVID and the mental and physical health of children and young people: national matched cohort study protocol (the CLoCk study)

**DOI:** 10.1136/bmjopen-2021-052838

**Published:** 2021-08-26

**Authors:** Terence Stephenson, Roz Shafran, Bianca De Stavola, Natalia Rojas, Felicity Aiano, Zahin Amin-Chowdhury, Kelsey McOwat, Ruth Simmons, Maria Zavala, CLoCk Consortium, Shamez N Ladhani, CLoCk Consortium members, Olivia Swann

**Affiliations:** 1 Population, Policy and Practice, UCL Great Ormond Street Institute of Child Health, London, UK; 2 Immunisation Department, Public Health England, London, UK

**Keywords:** COVID-19, paediatrics, infectious diseases, public health, virology

## Abstract

**Introduction:**

There is uncertainty surrounding the diagnosis, prevalence, phenotype, duration and treatment of Long COVID. This study aims to (A) describe the clinical phenotype of post-COVID symptomatology in children and young people (CYP) with laboratory-confirmed SARS-CoV-2 infection compared with test-negative controls, (B) produce an operational definition of Long COVID in CYP, and (C) establish its prevalence in CYP.

**Methods and analysis:**

A cohort study of SARS-CoV-2-positive CYP aged 11–17 years compared with age, sex and geographically matched SARS-CoV-2 test-negative CYP. CYP aged 11–17 testing positive and negative for SARS-CoV-2 infection will be identified and contacted 3, 6, 12 and 24 months after the test date. Consenting CYP will complete an online questionnaire. We initially planned to recruit 3000 test positives and 3000 test negatives but have since extended our target. Data visualisation techniques will be used to examine trajectories over time for symptoms and variables measured repeatedly, separately by original test status. Summary measures of fatigue and mental health dimensions will be generated using dimension reduction methods such as latent variables/latent class/principal component analysis methods. Cross-tabulation of collected and derived variables against test status and discriminant analysis will help operationalise preliminary definitions of Long COVID.

**Ethics and dissemination:**

Research Ethics Committee approval granted. Data will be stored in secure Public Health England servers or University College London’s Data Safe Haven. Risks of harm will be minimised by providing information on where to seek support. Results will be published on a preprint server followed by journal publication, with reuse of articles under a CC BY licence. Data will be published with protection against identification when there are small frequencies involved.

**Trial registration number:**

ISRCTN34804192; Pre-results.

**Strengths and limitations of this study**

- This study does not start with an arbitrary definition of a new condition—‘opinion-based medicine’.
- Rather it represents ‘evidence-based medicine’—we will seek the views of children and young people on what they have experienced physically and mentally in the months following COVID-19.
- The study incorporates a comparator matched cohort of children and young people who have experienced a pandemic, school closure and social isolation but who had a negative COVID-19 test.
- The comparator matched cohort of children and young people must also have had a reason to seek a COVID-19 test: symptoms, anxiety, a contact or a bereavement.
- Ideally, we would test all children for antibodies to confirm COVID-19 positive and negative status throughout the study period but we thought this would significantly reduce participation.

## Introduction

Currently, there is huge uncertainty surrounding the diagnosis, prevalence, phenotype, duration and treatment of Long COVID. There is currently no diagnostic test or code for Long COVID. Consequently, cases of Long COVID are not captured in routine National Health Service (NHS) administrative data sets. Instead, Long COVID may be coded as a variety of different conditions in hospital or symptom clusters in non-hospitalised children and young people (CYP). However, it is possible the symptoms associated with Long COVID are in fact a mixture of factors relating to the pandemic and lockdown as a whole rather than the viral infection itself. For example, factors such as social isolation, anxiety, depression or educational concerns may be the root cause of these symptoms in CYP both with and without SARS-CoV-2 infection. The effects on the developing brain and behaviour of adolescents could be far reaching.^1^

Despite acute COVID-19 illness being milder in CYP, it should not be assumed that those CYP at low risk of life-threatening acute infections^2 3^ do not suffer the longer term consequences of SARS-CoV-2 infection. It is important to study Long COVID within this population given that the psychological and social impact of Long COVID could have major consequences for transition to adulthood. There is a clear need to define the clinical phenotype of Long COVID in order to understand those most at risk, the illness trajectory, and to provide accurate information on the natural course of the condition. It is equally important to understand both the physical and mental health impact on CYP with Long COVID, given the rise in mental health problems among CYP since the start of the pandemic.^4^ Obtaining a better understanding of which CYP are affected will help with targeting potential interventions.

The greatest risk factor for SAR-CoV-2 illness has been old age, with other risk factors at all ages including obesity, comorbid long-term conditions, learning and neurological disabilities, mental health problems and ethnic minority status. It is plausible that such CYP may be most at risk of suffering from Long COVID, given that much is still unknown in CYP about the immunological susceptibility and underlying biology of Long COVID. Of all CYP, those likely to be most at risk of Long COVID are teenagers, with existing literature showing they make up the majority of CYP with chronic fatigue, post viral syndromes and persistent symptoms.^5^

In adults, there is emerging evidence that gender is a risk factor for Long COVID, with middle-aged females more susceptible than men.^6^ In terms of symptoms, two main groupings have been identified: (1) respiratory symptoms (eg, cough, shortness of breath) as well as fatigue and headaches, and (2) multisystem, including the brain, gut and heart. Among adults with Long COVID, heart symptoms such as palpitations or fast heartbeat, pins and needles or numbness and problems concentrating (‘brain fog’) have been reported. Experiencing a greater number of symptoms during the first week of infection, as well as older age, has also been linked to Long COVID. It should be noted mental health conditions were not reported in this cohort. Four syndromes have been described in adults: postviral fatigue; fluctuating multisystem symptoms; postintensive care syndrome; and lasting organ damage. It is known in both adults and young people that a wide range of long-term physical conditions increase the risk of mental ill health, particularly if the condition involves the central nervous system, with several studies reporting increased rates of all common mental health conditions in children.^7^ Moreover, fatigue has also been studied in paediatric long-term conditions including CYP with multiple sclerosis.^8 9^ Emerging clinical observations and preliminary research indicate that COVID-19 can have a long-term impact on CYP in a range of domains^10 11^ but well-conducted, methodologically robust studies are lacking.

This study primarily aims to (A) describe the clinical phenotype and prevalence of post-COVID-19 symptoms (eg, pain or physical symptoms, fatigue, sleep problems, mental health problems) among test-positive CYP compared to test-negative CYP, (B) produce an operational definition of Long COVID in CYP, and (C) establish the prevalence of Long COVID in CYP testing positive for SARS-CoV-2 infection. The second aim is of particular importance given that this is a prerequisite for any future epidemiological or interventional study. This will be achieved by using the empirical data we will acquire from 17 000 CYP and a national Delphi consensus process.

## Methods

### Study design and setting

Public Health England (PHE) has been conducting national surveillance of SARS-CoV-2 since the start of the pandemic in England. PHE receives daily electronic notifications of all SARS-CoV-2 RT-PCR tests performed in healthcare settings (Pillar 1 tests) and in the community (Pillar 2 tests), which are reported through the Second Generation Surveillance System (SGSS). Information within the SGSS reports includes NHS number, name, age, sex, postcode, date of sample, reporting laboratory and test result. PHE also has access to the electronic Patient Demographic Service (PDS), which contains the names, addresses and status (alive/dead) of all patients registered with the NHS.

A sample of CYP aged 11–17 years when testing positive for SARS-CoV-2 between September 2020 and March 2021 and COVID-19 test-negative CYP matched for age, sex and region identified through SGSS will be linked to PDS using available identifiers and postal addresses. A letter will be posted to them, informing them about the study and inviting them to take part using an online link. This link will provide them with information about the study, with an option to consent online and complete a short recruitment questionnaire (or both as paper options). Recruitment was planned to start in March 2021 and all data collection anticipated to end in April 2023.

This will be a cohort study of SARS-CoV-2 test-positive CYP aged 11–17 years matched on the test date, age, sex and geography to SARS-CoV-2 test-negative controls identified by PHE.

### Participants

Between September 2020 and March 2021, a total of 234 803 CYP aged between 11 and 17 years were diagnosed with COVID-19 in England. During the same period there were 1 481 154 negative tests among this age group equivalent to 1 203 996 CYP. Among those who tested negative 76 689 individuals (100 154 tests) were excluded as they had a positive result before and/or after their negative test. The positive and negative cohorts were linked using age at test, gender, month of test and lower super output area resulting in 102 402 positive individuals and 147 561 negative individuals. Matched individuals were checked against the NHS spine to exclude any individuals who have died, and to extract participants’ addresses to send invitation letters. Thirty-seven individuals were excluded because they had died since their COVID-19 test (six among those positive and 31 among those negative), and 11 193 individuals of the positive cohort and 19 251 individuals of the negative cohort were excluded as an address was not available. Finally, 187 CYP were excluded as they were included in a previous study. Following exclusions, 91 016 positive CYP and 128 220 negative CYP will be available to be invited to participate in the study. For all the months except December, all eligible individuals will be sent an invitation. For those tested in December, 2970 positive CYP and 5911 negative CYP will be invited as the eligible number was so large that additional funding would be needed to invite all those eligible.

**Figure 1:**
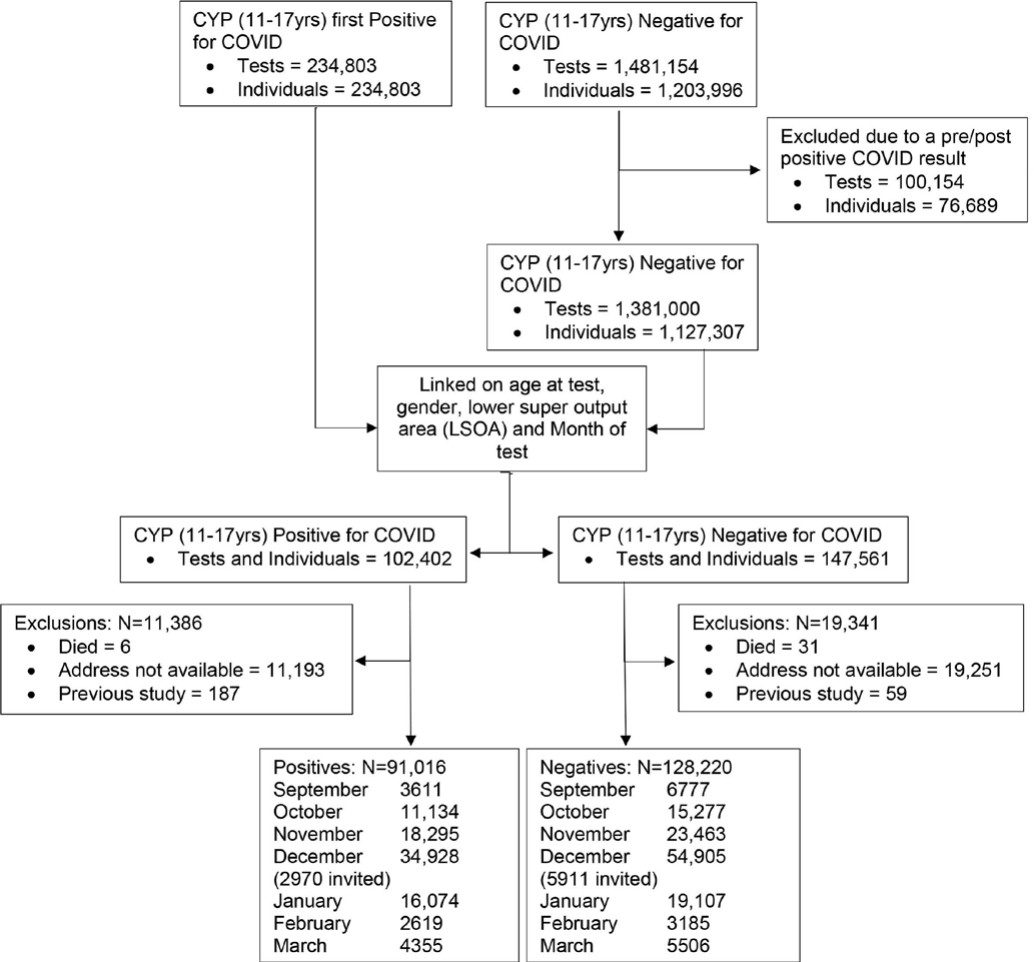
Flow chart of the selection process. CYP, children and young people.

This is shown schematically in figure 1.

### Data collection

Participating families of CYP will be contacted 3, 6, 12 and 24 months after the CYP’s SARS-CoV-2 test (depending on recruitment month) and invited to take part in the study. Following online informed consent, the CYP will self-complete an online (or paper) questionnaire about their mental and physical health. CYP towards the lower end of the 11–17 age band and CYP with special educational needs or disability may require the help of a carer to complete the questionnaire.

### Outcomes

The questionnaires given to CYP include elements of the International Severe Acute Respiratory and emerging Infection Consortium (ISARIC) Paediatric COVID-19 follow-up questionnaire^12^ and the recent Mental Health of Children and Young People in England surveys^13^ to facilitate international comparisons regarding the risk factors and profile of Long COVID in CYP. The questionnaires given to CYP were designed together with the ISARIC Paediatric Working Group to produce a harmonised data collection tool, allowing for standardisation and meta-analysis by design.

The elements taken from the ISARIC Paediatric COVID-19 follow-up questionnaire^12^ include questions about demographics and physical symptoms, with particular questions enquiring about cough and fever, given that these are the main acute symptoms in non-hospitalised CYP^14^ and gastrointestinal symptoms, as a study has shown such symptoms were common in seropositive CYP.^15^ It is likely other symptoms will manifest later in Long COVID (eg, tiredness, headaches, myalgia, etc), with the skin rashes and cardiac problems in hospitalised children with paediatric multisystem inflammatory syndrome being borne in mind for Long COVID.^5^ Those older CYP could very well overlap with adult symptomatology (eg, a neuropsychiatric-encephalitic subgroup).

In terms of the other questions, the Strengths and Difficulties Questionnaire^16^ will be used to assess emotional and mental health, consisting of 25 items. The EuroQol Group, 5-Dimension health-related quality of life instrument for Young people (EQ-5D-Y)^17^ will be used as a measure of quality of life/functioning, with fatigue measured using the 11-item Chalder Fatigue Questionnaire.^18^ Additionally, the short 7-item version of the Warwick-Edinburgh Mental Well-being Scale^19^ will be included. Loneliness will be measured using the 4-item UCLA (University of California at Los Angeles) Loneliness Scale for Children^20^ as well as self-reported school attendance.

The length of the online self-assessment has an estimated completion time of approximately 20 min for CYP. The CYP do not have to complete all of the questions at once but can pause and save the answers at any point.

### Statistical analyses

Response rates for positive and negative CYP will inform expectations of final study size and possibly refinement of the sampling percentages. Descriptive statistics will be used for all dimensions collected at baseline and the follow-up questionnaires, separately by test status, wave and month of test. Appropriate data visualisation techniques will be used to examine trajectories over time for those variables that are measured repeatedly, separately by test status and month of test. Summary measures of the fatigue and mental health dimensions will be generated using dimension reduction methods such as latent variables/latent class/principal component analysis methods. Cross-tabulation of latent variables against test status as well as discriminant analysis will help operationalise preliminary definitions of Long COVID. These will be compared with definitions based on categorisation of prolonged symptoms supplemented by a Delphi consensus process.

### Sample size

A total of 5000 participants (2500 test positive and 2500 test negative) would give 80% power to detect at least a 4% difference in symptoms at 5% significance, if the COVID-19-negative participants had a prevalence similar to that reported for adults (ie, 34%). Greater baseline prevalences (eg, 40%) would require slightly greater numbers for the same power and confidence; smaller baseline prevalences (eg, 20%) smaller numbers. However, identifying risk factors for Long COVID-19 would require studying demographic, social, family and clinical data and thus we would require larger numbers. For this reason, we are planning to invite all available participants.

### Patient and public involvement

Resources have been allocated for any patient and public involvement (PPI) activities at INVOLVE (a national advisory body funded by the National Institute for Health Research to support public involvement in NHS, public health and social care research and development) rates including a nationally representative PPI research advisory group. PPI members will be offered training and support in accordance with the INVOLVE^21^ report and other guidance^22^ including online training. PPI meetings will take place quarterly.

## Ethics and dissemination

### Ethical considerations

PHE has legal permission, provided by Regulation 3 of The Health Service (Control of Patient Information) Regulations 2002, to process patient confidential information for national surveillance of communicable diseases. Individual patient consent is not required for initial invitation to the study. Parents/carers and young people will be sent an invitation with a link to the website with the relevant information sheets and consent forms. They will have the opportunity to ask any questions about the study. Parents/carers of CYP under 16 years of age will be asked to complete a parent/carer consent form. The young person will also be asked to sign an assent form to indicate their agreement. Consent will be asked from 16–17 year-olds (using the Young Person Consent Form) but their parents will not. This is in line with Health Research Authority recommended processes.^23^

The study was approved by Yorkshire and the Humber–South Yorkshire Research Ethics Committee (REC reference: 21/YH/0060; IRAS project ID: 293495).

It is possible that the questionnaires may make some vulnerable participants feel fatigued or distressed from completing questionnaires relating to their mental health and/or report serious symptoms that put them at immediate risk. The research team will provide information on where to seek support and provide self-help information. Unfortunately, the researchers are unable to provide medical advice. However, existing national surveys of children’s mental health also follow this risk protocol.

### Dissemination

Peer-reviewed publications, briefings for policymakers and lay summaries for participants and CYP and carers. Results will be made public, initially on a preprint server, and we will allow reuse of articles under a CC BY licence. The data set will be made publicly available.

## Supplementary Material

Reviewer comments

Author's manuscript
