# Supplementary material for: Long COVID and the mental and physical health of children and young people: national matched cohort study protocol (the CLoCk study)
Source: BMJ Open. 2021 Aug 26;11(8):e052838. doi: 10.1136/bmjopen-2021-052838 (PMC8392739; doi:10.1136/bmjopen-2021-052838)
Supplement: Author's manuscript [file bmjopen-2021-052838.draft_revisions.pdf]

BMJ Open is committed to open peer review. As part of this commitment we make the peer review history of every article we publish publicly available.

When an article is published we post the peer reviewers' comments and the authors' responses online. We also post the versions of the paper that were used during peer review. These are the versions that the peer review comments apply to.

The versions of the paper that follow are the versions that were submitted during the peer review process. They are not the versions of record or the final published versions. They should not be cited or distributed as the published version of this manuscript.

BMJ Open is an open access journal and the full, final, typeset and author-corrected version of record of the manuscript is available on our site with no access controls, subscription charges or pay-per-view fees (http://bmjopen.bmj.com).

If you have any questions on BMJ Open's open peer review process please email info.bmjopen@bmj.com

BMJ Open

Long Covid and the mental and physical health of children and young people; a national matched cohort study (CLoCk).

Journal:	*BMJ Open*
Manuscript ID	bmjopen-2021-052838
Article Type:	Protocol
Date Submitted by the Author:	06-May-2021
Complete List of Authors:	Stephenson, Terence; UCL Great Ormond Street Institute of Child Health Population Policy and Practice<br>Shafran, Roz; UCL Great Ormond Street Institute of Child Health Population Policy and Practice<br>De Stavola, Bianca ; UCL Great Ormond Street Institute of Child Health Population Policy and Practice<br>Rojas, Natalia; UCL Great Ormond Street Institute of Child Health Population Policy and Practice<br>Aiano, Felicity; Public Health England<br>Amin-Chowdhury, Zahin; Public Health England<br>McOwat, Kelsey; Public Health England<br>Simmons, Ruth; Public Health England<br>Zavala, Maria; Public Health England<br>CLoCk, Consortium; UCL Great Ormond Street Institute of Child Health Population Policy and Practice<br>Ladhani, Shamez N.; Public Health England
Keywords:	COVID-19, PAEDIATRICS, INFECTIOUS DISEASES, PUBLIC HEALTH, VIROLOGY

SCHOLARONE™
Manuscripts

I, the Submitting Author has the right to grant and does grant on behalf of all authors of the Work (as defined in the below author licence), an exclusive licence and/or a non-exclusive licence for contributions from authors who are: i) UK Crown employees; ii) where BMJ has agreed a CC-BY licence shall apply, and/or iii) in accordance with the terms applicable for US Federal Government officers or employees acting as part of their official duties; on a worldwide, perpetual, irrevocable, royalty-free basis to BMJ Publishing Group Ltd ("BMJ") its licensees and where the relevant Journal is co-owned by BMJ to the co-owners of the Journal, to publish the Work in this journal and any other BMJ products and to exploit all rights, as set out in our licence.

The Submitting Author accepts and understands that any supply made under these terms is made by BMJ to the Submitting Author unless you are acting as an employee on behalf of your employer or a postgraduate student of an affiliated institution which is paying any applicable article publishing charge ("APC") for Open Access articles. Where the Submitting Author wishes to make the Work available on an Open Access basis (and intends to pay the relevant APC), the terms of reuse of such Open Access shall be governed by a Creative Commons licence – details of these licences and which Creative Commons licence will apply to this Work are set out in our licence referred to above.

Other than as permitted in any relevant BMJ Author's Self Archiving Policies, I confirm this Work has not been accepted for publication elsewhere, is not being considered for publication elsewhere and does not duplicate material already published. I confirm all authors consent to publication of this Work and authorise the granting of this licence.

For peer review only

Long Covid and the mental and physical health of children and young people; a national matched cohort study (CLoCk).

Abstract 250 words. Main paper 2294 words.

Stephenson T[1], Shafran R[1], De Stavola B[1], Rojas N[1], Aiano F[2], Amin-Chowdhury Z[2], McOwat K[2], Simmons R[2], Zavala M[2], CLoCk Consortium[1], Ladhani S[2].

1 UCL Great Ormond Street Institute of Child Health
2 Public Health England, Colindale

Corresponding author: Professor Sir Terence Stephenson; UCL Great Ormond Street Institute of Child Health, 30 Guilford Street, London, WC1N 1EH; Email: t.stephenson@ucl.ac.uk; Telephone: 020 7242 9789; Fax Number: N/A

Five Keywords/phrases: Long COVID, children, young people, cohort study, physical and mental health

For peer review only

For peer review only - http://bmjopen.bmj.com/site/about/guidelines.xhtml

Abstract

Introduction: There is uncertainty surrounding the diagnosis, prevalence, phenotype, duration and treatment of Long COVID. This study aims to (a) describe the clinical phenotype of post-COVID symptomatology in CYP with laboratory-confirmed SARS-CoV-2 infection compared to test-negative controls (b) produce an operational definition of Long COVID in CYP (c) establish the prevalence of Long COVID in CYP.

Methods and analysis: A cohort study of SARS-CoV-2 positive CYP aged 11-17 years compared with age-, sex- and geographically-matched SARS-CoV-2 test-negative CYP. CYP aged 11-17 testing positive and negative for SARS-CoV-2 infection will be identified and contacted 3, 6, 12 and 24 months after the test date. Consenting CYP will complete an online questionnaire. We will recruit 3,000 test positives and 3,000 test negatives. Data visualisation techniques will be used to examine trajectories over time for symptoms and variables measured repeatedly, separately by original test status. Summary measures of fatigue and mental health dimensions will be generated using dimension reduction methods such as latent variables/latent class/PCA methods. Cross-tabulation of collected and derived variables against test status and discriminant analysis will help operationalise preliminary definitions of Long COVID.

Ethics and Dissemination: Research Ethics Committee Approval granted. Data will be stored in secure PHE servers or UCL's Data Safe Haven. Risks of harm will be minimised by providing information on where to seek support. Results will be published on a pre-print server followed by journal publication, with reuse of articles under a CC BY licence. Data will be published with protection against identification when small frequencies.

Study registration: Study registered with ISRCTN: Trial ID: ISRCTN34804192 and granted Urgent Public Health prioritisation.

Data Sharing: Applications for relevant anonymised data should be submitted to the Public Health England office for Data Release: https://www.gov.uk/government/publications/accessing-public-health-england-data/about-the-phe-odr-and-accessing-data

For peer review only - http://bmjopen.bmj.com/site/about/guidelines.xhtml

Article Summary

STRENGTHS

- This study does not start with an arbitrary definition of a new condition – 'opinion based medicine'
- Rather it represents 'evidence based medicine' - we will seek the views of 6,000 children and young people on what they have experienced physically and mentally in the months following COVID-19
- The study incorporates a comparator matched cohort of children and young people who have experienced a pandemic, school closure and social isolation but who had a negative COVID test

WEAKNESSES

- The comparator matched cohort of children and young people must also have had a reason to seek a COVID test: symptoms, anxiety, a contact or a bereavement
- Ideally, we would test all children for antibodies to confirm COVID-19 positive and negative status throughout the study period but we thought would significantly reduce participation

For peer review only - http://bmjopen.bmj.com/site/about/guidelines.xhtml

Introduction

Currently, there is huge uncertainty surrounding the diagnosis, prevalence, phenotype, duration and treatment of Long COVID. There is currently no diagnostic test or code for Long COVID. Consequently, cases of Long COVID are not captured in routine National Health Service (NHS) administrative datasets. Instead, Long COVID may be coded as a variety of different conditions in hospital or symptom clusters in non-hospitalised CYP. However, it is possible the symptoms associated with Long COVID are in fact a mixture of factors relating to the pandemic and lockdown as a whole rather than the viral infection itself. For example, factors such as social isolation, anxiety, depression or educational concerns may be the root cause of these symptoms in CYP both with and without SARS-CoV-2 infection. The effects on the developing brain and behaviour of adolescents could be far reaching[1].

Despite acute COVID-19 illness being milder in CYP, it should not be assumed that those CYP at low risk of life-threatening acute infections[2 3] do not suffer the longer-term consequences of SARS-CoV-2 infection. It is important to study Long COVID within this population given that the psychological and social impact of Long COVID could have major consequences for transition to adulthood. There is a clear need to define the clinical phenotype of Long COVID in order to understand those most at risk, the illness trajectory and to provide accurate information on the natural course of the condition. It is equally important to understand both the physical and mental health impact on CYP with Long COVID, given the rise in mental health problems among CYP since the start of the pandemic[4]. Obtaining a better understanding of which CYP are affected will help with targeting potential interventions.

The greatest risk factor for severe acute SAR-CoV-2 illness has been old age, with other risk factors at all ages including obesity, comorbid long-term conditions, learning and neurological disabilities, mental health problems and ethnic minority status. It is plausible that such CYP may be most at risk of suffering from Long COVID, given that much is still unknown in CYP about the immunological susceptibility and underlying biology of Long COVID. Of all CYP, those likely to be most at risk of Long COVID are teenagers, with existing literature showing they make up the majority of CYP with chronic fatigue, post viral syndromes and persistent symptoms[5].

In adults, there is emerging evidence that gender is a risk factor for Long COVID, with middle-aged females more susceptible than men[6]. In terms of symptoms, two main groupings have been identified (i) respiratory symptoms (e.g. cough, shortness of breath) as well as fatigue and headaches, and (ii) multi-system, including the brain, gut and heart. Among adults with Long COVID, heart symptoms such as palpitations or fast heartbeat, pins and needles or numbness and problems concentrating (‘brain fog’) have been reported. Experiencing a greater number of symptoms during the first week of infection, as well as older age, have also been linked to Long COVID. It should be noted mental health conditions were not reported in this cohort. Four syndromes have been described in adults: Post-viral fatigue; Fluctuating multi-system symptoms; Post-intensive care syndrome; and Lasting organ damage. It is known in both adults and young people that a wide range of long-term physical conditions increase the risk of mental ill-health, particularly if the condition involves the central nervous system, with several studies

For peer review only - http://bmjopen.bmj.com/site/about/guidelines.xhtml

reporting increased rates of all common mental health conditions in children[7]. Moreover, fatigue has also been studied in paediatric long-term conditions including CYP with multiple sclerosis[8 9]. Emerging clinical observations and preliminary research indicate that COVID-19 can have a long-term impact on CYP in a range of domains[10 11] but well conducted, methodologically robust studies are lacking.

This study primarily aims to (a) describe the clinical phenotype and prevalence of post-COVID symptoms (e.g. pain or physical symptoms, fatigue, sleep problems, mental health problems) among test positive and test negative CYP, (b) produce an operational definition of Long COVID in CYP, and (c) establish the prevalence of Long COVID in CYP testing positive for SARS-CoV-2 infection. The second aim is of particular importance given that this is a prerequisite for any future epidemiological or interventional study. This will be achieved by using the empirical data we will acquire from 6,000 CYP and a national Delphi consensus process.

Methods

Study design and setting

Public Health England (PHE) has been conducting national surveillance of SARS-CoV-2 since the start of the pandemic in England. PHE receives daily electronic notifications of all SARS-CoV-2 RT-PCR tests performed in healthcare settings (Pillar 1 tests) and in the community (Pillar 2 tests), which are reported through the Second Generation Surveillance System (SGSS). Information within the SGSS reports includes NHS number, name, age, sex, postcode, date of sample, reporting laboratory and test result. PHE also has access to the electronic Patient Demographic Service (PDS), which contains the names, addresses and status (alive/dead) of all patients registered with the NHS.

A sample of CYP aged 11-17 years old when testing positive for SARS-CoV-2 between September 2020 and March 2021 and twice as many COVID test-negative CYP matched for age, sex and region identified through SGSS will be linked to PDS using available identifiers and postal addresses. A letter will be posted to them, informing them about the study and inviting them to take part using an online link. This link will provide them with information about the study, with an option to consent online and complete a short recruitment questionnaire (or both as paper options).

This will be a cohort study of SARS-CoV-2 test-positive CYP aged 11-17 years matched on the test date, age, sex and geography to SARS-CoV-2 test-negative controls identified by PHE.

Participants

Between 1st September 2020 and 31st March 2021, 234,803 CYP aged between 11 and 17 years were tested positive for SARS-CoV-2 in England, and 1,245,288 CYP tested negative. Among those who tested negative, 80,365 were excluded as they had a positive result pre- and/or post their negative test and we wish to compare test-positive with test-negative CYP. The positive and

For peer review only - http://bmjopen.bmj.com/site/about/guidelines.xhtml

negative cohorts were matched using age at test, gender, month of test, and lower super output area resulting in 102,402 positive individuals and 147,561 negative individuals. Matched individuals were checked against PDS to exclude any individuals who have died, and to extract participants addresses to send invitation letters. 33 individuals were excluded because of death since their COVID-19 test (6 among those positive and 27 among those negative), and 11,982 individuals of the positive cohort and 16,635 individuals of the negative cohort were excluded as an address was not available. Finally, 246 CYP were excluded as they were included in a previous pilot study which helped inform our methodology. Following exclusions, 90,227 positive CYP and 130,243 negative CYP form the population from which we will randomly sampled individuals for inclusion within the study.

Participants will be contacted in a ratio of 1:2 positives:negatives on the expectation of a lower participation rate in the latter. We anticipate 20% to be recruited in the positive group, as estimated by CYP recruitment rates in other PHE studies on COVID-19 (sKIDs)[12]. We also oversampled CYP who had a test in the months of January-March 2021 because only for these groups we will collect information at 3 months of follow-up.

This is shown schematically in Figure 1.

Data collection

Participating families of CYP will be contacted 3, 6, 12 and 24 months after the CYP's SARS-CoV-2 test (depending on recruitment month) and invited to take part in the study. Following online informed consent, the CYP will self-complete an online (or paper) questionnaire about their mental and physical health. CYP towards the lower end of the 11-18 age band and CYP with special educational needs or disability may require the help of a carer to complete the questionnaire.

Outcomes

The questionnaires given to CYP include elements of the ISARIC Paediatric COVID-19 follow-up questionnaire[13] and the recent Mental Health of Children and Young people in England surveys[14] to facilitate international comparisons regarding the risk factors and profile of Long COVID in CYP. The questionnaires given to CYP were designed together with the International Severe Acute Respiratory and emerging Infection Consortium (ISARIC) Paediatric Working Group to produce a harmonised data collection tool, allowing for standardisation and metanalysis by design.

The elements taken from the ISARIC Paediatric COVID-19 follow-up questionnaire[13] include questions about demographics and physical symptoms, with particular questions enquiring about cough and fever, given that these are the main acute symptoms in non-hospitalised CYP[15] and gastrointestinal symptoms, as a study has shown such symptoms were common in seropositive CYP[16]. It is likely other symptoms will manifest later in Long COVID (e.g., tiredness, headaches, myalgia etc.), with the skin rashes and cardiac problems in hospitalised children

For peer review only - http://bmjopen.bmj.com/site/about/guidelines.xhtml

with Paediatric multisystem inflammatory syndrome being borne in mind for Long COVID[5]. Those older CYP could very well overlap with adult symptomatology (e.g., a neuropsychiatric-encephalitic sub-group).

In terms of the other questions, the Strengths and Difficulties Questionnaire (SDQ)[17] will be used to assess emotional and mental health, consisting of 25 items. The EQ-5D-Y[18] will be used as a measure of quality of life/functioning, with fatigue measured using the 11-item Chalder Fatigue Questionnaire (CFQ)[19]. Additionally, the short, 7-item version of The Warwick Edinburgh Mental Wellbeing Scale (WEMWBS)[20] will be included. Loneliness will be measured using the 4-item UCLA Loneliness Scale for Children[21] as well as self-reported school attendance.

The length of the online self-assessment has an estimated completion time of approximately 20 minutes for CYP. The CYP do not have to complete all of the questions at once but can pause and save the answers at any point.

Statistical analyses

Response rates for positive and negative CYP will inform expectations of final study size and possibly refinement of the sampling percentages. Descriptive statistics will be used for all dimensions collected at baseline and the follow-up questionnaires, separately by test status, wave and month of test. Appropriate data visualisation techniques will be used to examine trajectories over time for those variables that are measured repeatedly, separately by test status and month of test. Summary measures of the fatigue and mental health dimensions will be generated using dimension reduction methods such as latent variables/latent class/PCA methods. Cross-tabulation of latent variables against test status as well as discriminant analysis will help operationalise preliminary definitions of Long COVID. These will be compared with definitions based on categorisation of prolonged symptoms supplemented by a Delphi consensus process.

Sample size

A total of 5,000 participants (2,500 test-positive and 2,500 test-negative) would give 80% power to detect at least a 4% difference in symptoms at 5% significance, if the COVID-19 negative participants had a prevalence similar to that reported for adults (i.e., 34%). Greater baseline prevalences (e.g., 40%) would require slightly greater numbers for the same power and confidence; smaller baseline prevalences (e.g., 20%) smaller numbers. However, identifying risk factors for long COVID-19 would require studying demographic, social, family and clinical data and thus we would require larger numbers. For this reason, we are targeting the recruitment of 6,000 participants.

For peer review only - http://bmjopen.bmj.com/site/about/guidelines.xhtml

Patient and Public Involvement

Resources have been allocated for any Patient and Public Involvement (PPI) activities at INVOLVE rates including a nationally representative PPI research advisory group. PPI members will be offered training and support in accordance with the INVOLVE[22] report and other guidance[23] including online training. PPI meetings will take place quarterly.

Ethics and Dissemination

Ethical considerations

PHE has legal permission, provided by Regulation 3 of The Health Service (Control of Patient Information) Regulations 2002, to process patient confidential information for national surveillance of communicable diseases. Individual patient consent is not required for initial invitation to the study. Parents/carers and young people will be sent an invitation with a link to the website with the relevant information sheets and consent forms. They will have the opportunity to ask any questions about the study. Parents/carers of CYP under 16 years of age will be asked to complete a Parent/carer consent form. The young person will also be asked to sign an assent form to indicate their agreement. 16-18-year-olds will be asked for consent (using the Young Person Consent Form); but their parents will not. This is in line with HRA recommended processes[24].

Study approved by Yorkshire & The Humber - South Yorkshire Research Ethics Committee (REC reference: 21/YH/0060; IRAS project ID:293495).

It is possible that the questionnaires may make some vulnerable participants feel fatigued or distressed from completing questionnaires relating to their mental health and/or report serious symptoms that put them at immediate risk. The research team will provide information on where to seek support and provide self-help information. Unfortunately, the researchers are unable to provide medical advice. However, existing national surveys of children's mental health also follow this risk protocol.

Dissemination

Peer reviewed publications, briefings for policy makers and lay summaries for participants and CYP and carers. Results will be made public, initially on a pre-print server and we will allow reuse of articles under a CC BY licence. The dataset will be made publicly available.

For peer review only

Author contributions:

Terence Stephenson t.stephenson@ucl.ac.uk conceived the idea for the study, submitted the successful grant application and undertook the first revision of the manuscript.
Roz Shafran r.shafran@ucl.ac.uk provided ideas on mental health follow up to the original grant application, submitted the ethics and R&D applications and revised the manuscript.
Bianca De Stavola b.destavola@ucl.ac.uk provided statistical input to the design and analysis, including sample size calculations.
Natalia Rojas n.rojas@ucl.ac.uk wrote the first draft of the manuscript based on the grant application, protocol and ethics form and compiled the references.
Felicity Aiano Felicity.Aiano@phe.gov.uk helped develop the study design
Zahin Amin-Chowdhury Zahin.Amin@phe.gov.uk helped develop of the study design
Kelsey McOwat Kelsey.Mcowat@phe.gov.uk adapted the questionnaire for the online SNAP survey platform.
Maria Zavala Maria.Zavala@phe.gov.uk undertook a separate pilot study which informed the CLoCk consent process and the online questionnaire.
Ruth Simmons Ruth.Simmons@phe.gov.uk designed the participant sampling and dataflow.
Shamez Ladhani shamez.ladhani@phe.gov.uk operationalised the regulatory and recruitment ideas for the study and revised the manuscript.

Olivia Swann and Elizabeth Whittaker designed the elements of the ISARIC Paediatric COVID-19 follow-up questionnaire which were incorporated into the online questionnaire used in this study to which all the CLoCk Consortium members contributed.

All members of the CLoCk Consortium made substantial contributions to the conception or design of the work; were involved in drafting both the funding application and this manuscript; approved the version to be published; and agree to be accountable for all aspects of the work in ensuring that questions related to the accuracy or integrity of any part of the work are appropriately investigated and resolved.

Co-Applicants on the grant application and CLoCk Consortium members (alphabetical):

Marta Buszewicz, University College London
Trudie Chalder, King's College London, Chalder, trudie.chalder@kcl.ac.uk
Esther Crawley, University of Bristol, Esther.Crawley@bristol.ac.uk
Emma Dalrymple, PPI representative, emmajdalrymple@gmail.com
Tamsin Jane Ford, University of Cambridge, tjf52@medschl.cam.ac.uk
Shruti Garg, University of Manchester, Shruti.Garg@mft.nhs.uk
Anthony Harnden, University of Oxford, anthony.harnden@phc.ox.ac.uk
Dougal Hargreaves, Imperial College London, Dougal.HARGREAVES@education.gov.uk
Isobel Heyman, Great Ormond Street Hospital for Children NHS Foundation Trust Heyman, i.heyman@ucl.ac.uk,
Michael Levin, Imperial College London, m.levin@imperial.ac.uk
Vanessa Poustie, University of Liverpool, v.poustie@liverpool.ac.uk
Terry Segal, University College London Hospitals NHS Foundation Trust, terry.segal@nhs.net
Malcolm Semple, University of Liverpool, M.G.Semple@liverpool.ac.uk

For peer review only - http://bmjopen.bmj.com/site/about/guidelines.xhtml

Kishan Sharma, Manchester University NHS Foundation Trust, Kishan.Sharma@mft.nhs.uk
Olivia Swann, University of Edinburgh, olivia.swann@ed.ac.uk
Elizabeth Whittaker, Imperial College London, e.whittaker@imperial.ac.uk

Acknowledgement: Michael Lattimore, Public Health England, as Project Officer for the CLoCk study

Competing interests statement: Terence Stephenson is Chair of the Health Research Authority and therefore recused himself from the Research Ethics Application

Funding statement: Funded by The Department of Health and Social Care, in their capacity as the National Institute for Health Research (NIHR), and by UK Research & Innovation (UKRI) who have awarded funding grant number COVLT0022. All research at Great Ormond Street Hospital NHS Foundation Trust and UCL Great Ormond Street Institute of Child Health is made possible by the NIHR Great Ormond Street Hospital Biomedical Research Centre. The views expressed are those of the author(s) and not necessarily those of the NHS, the NIHR or the Department of Health

For peer review only - http://bmjopen.bmj.com/site/about/guidelines.xhtml

References

1. Orben A, Tomova L, Blakemore S-J. The effects of social deprivation on adolescent development and mental health. *The Lancet Child & Adolescent Health* 2020;4(8):634-40. doi: 10.1016/s2352-4642(20)30186-3
2. Whittaker E, Bamford A, Kenny J, et al. Clinical Characteristics of 58 Children With a Pediatric Inflammatory Multisystem Syndrome Temporally Associated With SARS-CoV-2. *JAMA* 2020;324(3):259-69. doi: 10.1001/jama.2020.10369 [published Online First: 2020/06/09]
3. Swann OV, Holden KA, Turtle L, et al. Clinical characteristics of children and young people admitted to hospital with covid-19 in United Kingdom: prospective multicentre observational cohort study. *BMJ* 2020;370:m3249. doi: 10.1136/bmj.m3249 [published Online First: 2020/09/23]
4. Pierce M, Hope H, Ford T, et al. Mental health before and during the COVID-19 pandemic: a longitudinal probability sample survey of the UK population. *The Lancet Psychiatry* 2020;7(10):883-92. doi: 10.1016/s2215-0366(20)30308-4
5. Ludvigsson JF. Case report and systematic review suggest that children may experience similar long-term effects to adults after clinical COVID-19. *Acta Paediatr* 2021;110(3):914-21. doi: 10.1111/apa.15673 [published Online First: 2020/11/19]
6. Sudre CH, Murray B, Varsavsky T, et al. Attributes and predictors of Long-COVID: analysis of COVID cases and their symptoms collected by the Covid Symptoms Study App. 2020 doi: 10.1101/2020.10.19.20214494
7. Moore DA, Nunns M, Shaw L, et al. Interventions to improve the mental health of children and young people with long-term physical conditions: linked evidence syntheses. *Health Technol Assess* 2019;23(22):1-164. doi: 10.3310/hta23220 [published Online First: 2019/05/28]
8. Carroll S, Chalder T, Hemingway C, et al. "It feels like wearing a giant sandbag." Adolescent and parent perceptions of fatigue in paediatric multiple sclerosis. *Eur J Paediatr Neurol* 2016;20(6):938-45. doi: 10.1016/j.ejpn.2016.06.004 [published Online First: 2016/07/17]
9. Carroll S, Chalder T, Hemingway C, et al. Understanding fatigue in paediatric multiple sclerosis: a systematic review of clinical and psychosocial factors. *Dev Med Child Neurol* 2016;58(3):229-39. doi: 10.1111/dmcn.12964 [published Online First: 2015/11/15]
10. Buonsenso D, Espuny Pujol F, Munblit D, et al. Clinical Characteristics, Activity Levels and Mental Health Problems in Children with Long COVID: A Survey of 510 Children. *Preprints* 2021 doi: 10.20944/preprints202103.0271.v1
11. Munblit D, De Rose C, Sinatti D, et al. Preliminary Evidence on Long COVID in children. *medRxiv* 2021
12. S L. Prospective active national surveillance of preschools and primary schools for SARS-CoV-2 infection and transmission in England, June 2020 (sKIDs COVID-19 surveillance in school KIDs). 2020 [Available from: https://tinyurl.com/sKIDsPhase1Report accessed 13 April 2021.
13. Sigfrid L, Maskell K, Bannister PG, et al. Addressing challenges for clinical research responses to emerging epidemics and pandemics: a scoping review. *BMC Med* 2020;18(1):190. doi: 10.1186/s12916-020-01624-8 [published Online First: 2020/06/27]
14. NHS Digital. Mental Health of Children and Young People in England, 2020: Wave 1 follow up to the 2017 survey 2020 [Available from: https://tinyurl.com/NHSCYPFU17 accessed 13 April 2021.

For peer review only - http://bmjopen.bmj.com/site/about/guidelines.xhtml

15. Ladhani SN, Amin-Chowdhury Z, Davies HG, et al. COVID-19 in children: analysis of the first pandemic peak in England. *Arch Dis Child* 2020;105(12):1180-85. doi: 10.1136/archdischild-2020-320042 [published Online First: 2020/08/17]
16. Waterfield T, Watson C, Moore R, et al. Seroprevalence of SARS-CoV-2 antibodies in children: a prospective multicentre cohort study. *Arch Dis Child* 2020 doi: 10.1136/archdischild-2020-320558 [published Online First: 2020/11/12]
17. Goodman R. Psychometric properties of the strengths and difficulties questionnaire. *J Am Acad Child Adolesc Psychiatry* 2001;40(11):1337-45. doi: 10.1097/00004583-200111000-00015 [published Online First: 2001/11/09]
18. Wille N, Badia X, Bonsel G, et al. Development of the EQ-5D-Y: a child-friendly version of the EQ-5D. *Qual Life Res* 2010;19(6):875-86. doi: 10.1007/s11136-010-9648-y [published Online First: 2010/04/21]
19. Chalder T, Berelowitz G, Pawlikowska T, et al. Development of a fatigue scale. *J Psychosom Res* 1993;37(2):147-53. doi: 10.1016/0022-3999(93)90081-p [published Online First: 1993/01/01]
20. Tennant R, Hiller L, Fishwick R, et al. The Warwick-Edinburgh Mental Well-being Scale (WEMWBS): development and UK validation. *Health Qual Life Outcomes* 2007;5:63. doi: 10.1186/1477-7525-5-63 [published Online First: 2007/11/29]
21. Office of National Statistics. Children's and young people's experiences of loneliness: 2018 2018 [Available from: https://tinyurl.com/CYPExperiencesOfLoneliness accessed 13 April 2021.
22. INVOLVE. Developing training and support for public involvement in research 2012 [Available from: https://www.invo.org.uk/wp-content/uploads/2012/11/INVOLVETrainingSupport2012.pdf accessed 13 April 2020.
23. National Institute for Health Research (NIHR). PPI (Patient and Public Involvement) resources for applicants to NIHR research programmes. 2019 [Available from: https://tinyurl.com/PPIApplicantResources accessed 13 April 2021.
24. Health Research Authority (HRA). Research involving children 2018 [Available from: https://www.hra.nhs.uk/planning-and-improving-research/policies-standards-legislation/research-involving-children/ accessed 13 April 2021.

For peer review only - http://bmjopen.bmj.com/site/about/guidelines.xhtml

Figure 1: Flowchart of the selection process.

For peer review only

For peer review only - http://bmjopen.bmj.com/site/about/guidelines.xhtml

Figure 1: Flowchart of the selection process.

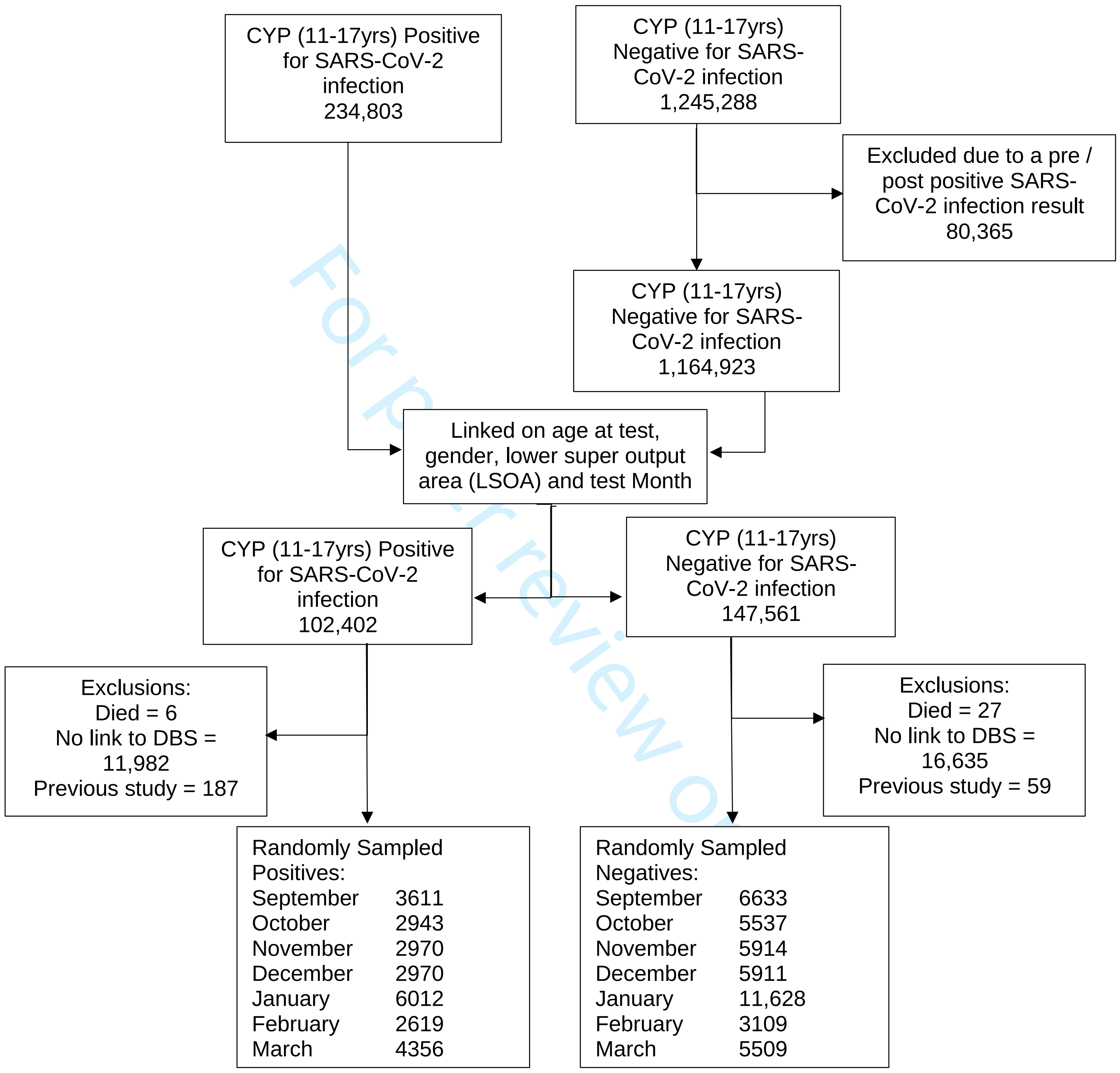

For peer review only - http://bmjopen.bmj.com/site/about/guidelines.xhtml

BMJ Open

Long Covid and the mental and physical health of children and young people: national matched cohort (CLoCk) study protocol

Journal:	*BMJ Open*
Manuscript ID	bmjopen-2021-052838.R1
Article Type:	Protocol
Date Submitted by the Author:	09-Jul-2021
Complete List of Authors:	Stephenson, Terence; UCL Great Ormond Street Institute of Child Health Population Policy and Practice<br>Shafran, Roz; UCL Great Ormond Street Institute of Child Health Population Policy and Practice<br>De Stavola, Bianca ; UCL Great Ormond Street Institute of Child Health Population Policy and Practice<br>Rojas, Natalia; UCL Great Ormond Street Institute of Child Health Population Policy and Practice<br>Aiano, Felicity; Public Health England<br>Amin-Chowdhury, Zahin; Public Health England<br>McOwat, Kelsey; Public Health England<br>Simmons, Ruth; Public Health England<br>Zavala, Maria; Public Health England<br>CLoCk, Consortium; UCL Great Ormond Street Institute of Child Health Population Policy and Practice<br>Ladhani, Shamez N.; Public Health England
**Primary Subject Heading**:	Paediatrics
Secondary Subject Heading:	Public health, Infectious diseases
Keywords:	COVID-19, PAEDIATRICS, INFECTIOUS DISEASES, PUBLIC HEALTH, VIROLOGY

SCHOLARONE™
Manuscripts

I, the Submitting Author has the right to grant and does grant on behalf of all authors of the Work (as defined in the below author licence), an exclusive licence and/or a non-exclusive licence for contributions from authors who are: i) UK Crown employees; ii) where BMJ has agreed a CC-BY licence shall apply, and/or iii) in accordance with the terms applicable for US Federal Government officers or employees acting as part of their official duties; on a worldwide, perpetual, irrevocable, royalty-free basis to BMJ Publishing Group Ltd ("BMJ") its licensees and where the relevant Journal is co-owned by BMJ to the co-owners of the Journal, to publish the Work in this journal and any other BMJ products and to exploit all rights, as set out in our licence.

The Submitting Author accepts and understands that any supply made under these terms is made by BMJ to the Submitting Author unless you are acting as an employee on behalf of your employer or a postgraduate student of an affiliated institution which is paying any applicable article publishing charge ("APC") for Open Access articles. Where the Submitting Author wishes to make the Work available on an Open Access basis (and intends to pay the relevant APC), the terms of reuse of such Open Access shall be governed by a Creative Commons licence – details of these licences and which Creative Commons licence will apply to this Work are set out in our licence referred to above.

Other than as permitted in any relevant BMJ Author's Self Archiving Policies, I confirm this Work has not been accepted for publication elsewhere, is not being considered for publication elsewhere and does not duplicate material already published. I confirm all authors consent to publication of this Work and authorise the granting of this licence.

For peer review only

Long Covid and the mental and physical health of children and young people: national matched cohort (CLoCk) study protocol

Abstract 280 words. Main paper 2284 words.

Stephenson T[1], Shafran R[1], De Stavola B[1], Rojas N[1], Aiano F[2], Amin-Chowdhury Z[2], McOwat K[2], Simmons R[2], Zavala M[2], CLoCk Consortium[1], Ladhani S[2].

1 UCL Great Ormond Street Institute of Child Health
2 Public Health England, Colindale

Corresponding author: Professor Sir Terence Stephenson; UCL Great Ormond Street Institute of Child Health, 30 Guilford Street, London, WC1N 1EH; Email: t.stephenson@ucl.ac.uk; Telephone: 020 7242 9789; Fax Number: N/A

Five Keywords/phrases: Long COVID, children, young people, cohort study, physical and mental health

For peer review only

Abstract

Introduction: There is uncertainty surrounding the diagnosis, prevalence, phenotype, duration and treatment of Long COVID. This study aims to (a) describe the clinical phenotype of post-COVID symptomatology in children and young people (CYP) with laboratory-confirmed SARS-CoV-2 infection compared to test-negative controls, (b) produce an operational definition of Long COVID in CYP, and (c) establish its prevalence in CYP.

Methods and analysis: A cohort study of SARS-CoV-2 positive CYP aged 11-17 years compared with age-, sex- and geographically-matched SARS-CoV-2 test-negative CYP. CYP aged 11-17 testing positive and negative for SARS-CoV-2 infection will be identified and contacted 3, 6, 12 and 24 months after the test date. Consenting CYP will complete an online questionnaire. We initially planned to recruit 3,000 test positives and 3,000 test negatives but have since extended our target. Data visualisation techniques will be used to examine trajectories over time for symptoms and variables measured repeatedly, separately by original test status. Summary measures of fatigue and mental health dimensions will be generated using dimension reduction methods such as latent variables/latent class/PCA methods. Cross-tabulation of collected and derived variables against test status and discriminant analysis will help operationalise preliminary definitions of Long COVID.

Ethics and Dissemination: Research Ethics Committee Approval granted. Data will be stored in secure Public Health England (PHE) servers or University College London's Data Safe Haven. Risks of harm will be minimised by providing information on where to seek support. Results will be published on a pre-print server followed by journal publication, with reuse of articles under a CC BY licence. Data will be published with protection against identification when small frequencies.

Study registration: Study registered with ISRCTN: Trial ID: ISRCTN34804192 and granted Urgent Public Health prioritisation.

For peer review only - http://bmjopen.bmj.com/site/about/guidelines.xhtml

Article Summary

STRENGTHS

- This study does not start with an arbitrary definition of a new condition – 'opinion-based medicine'
- Rather it represents 'evidence-based medicine' - we will seek the views of children and young people on what they have experienced physically and mentally in the months following COVID-19
- The study incorporates a comparator matched cohort of children and young people who have experienced a pandemic, school closure and social isolation but who had a negative COVID test

WEAKNESSES

- The comparator matched cohort of children and young people must also have had a reason to seek a COVID test: symptoms, anxiety, a contact or a bereavement
- Ideally, we would test all children for antibodies to confirm COVID-19 positive and negative status throughout the study period but we thought would significantly reduce participation

For peer review only - http://bmjopen.bmj.com/site/about/guidelines.xhtml

Introduction

Currently, there is huge uncertainty surrounding the diagnosis, prevalence, phenotype, duration and treatment of Long COVID. There is currently no diagnostic test or code for Long COVID. Consequently, cases of Long COVID are not captured in routine National Health Service (NHS) administrative datasets. Instead, Long COVID may be coded as a variety of different conditions in hospital or symptom clusters in non-hospitalised children and young people (CYP). However, it is possible the symptoms associated with Long COVID are in fact a mixture of factors relating to the pandemic and lockdown as a whole rather than the viral infection itself. For example, factors such as social isolation, anxiety, depression or educational concerns may be the root cause of these symptoms in CYP both with and without SARS-CoV-2 infection. The effects on the developing brain and behaviour of adolescents could be far reaching[1].

Despite acute COVID-19 illness being milder in CYP, it should not be assumed that those CYP at low risk of life-threatening acute infections[2 3] do not suffer the longer-term consequences of SARS-CoV-2 infection. It is important to study Long COVID within this population given that the psychological and social impact of Long COVID could have major consequences for transition to adulthood. There is a clear need to define the clinical phenotype of Long COVID in order to understand those most at risk, the illness trajectory and to provide accurate information on the natural course of the condition. It is equally important to understand both the physical and mental health impact on CYP with Long COVID, given the rise in mental health problems among CYP since the start of the pandemic[4]. Obtaining a better understanding of which CYP are affected will help with targeting potential interventions.

The greatest risk factor for severe acute SAR-CoV-2 illness has been old age, with other risk factors at all ages including obesity, comorbid long-term conditions, learning and neurological disabilities, mental health problems and ethnic minority status. It is plausible that such CYP may be most at risk of suffering from Long COVID, given that much is still unknown in CYP about the immunological susceptibility and underlying biology of Long COVID. Of all CYP, those likely to be most at risk of Long COVID are teenagers, with existing literature showing they make up the majority of CYP with chronic fatigue, post viral syndromes and persistent symptoms[5].

In adults, there is emerging evidence that gender is a risk factor for Long COVID, with middle-aged females more susceptible than men[6]. In terms of symptoms, two main groupings have been identified (i) respiratory symptoms (e.g., cough, shortness of breath) as well as fatigue and headaches, and (ii) multi-system, including the brain, gut and heart. Among adults with Long COVID, heart symptoms such as palpitations or fast heartbeat, pins and needles or numbness and problems concentrating ('brain fog') have been reported. Experiencing a greater number of symptoms during the first week of infection, as well as older age, have also been linked to Long COVID. It should be noted mental health conditions were not reported in this cohort. Four syndromes have been described in adults: Post-viral fatigue; Fluctuating multi-system symptoms; Post-intensive care syndrome; and Lasting organ damage. It is known in both adults and young people that a wide range of long-term physical conditions increase the risk of mental ill-health, particularly if the condition involves the central nervous system, with several studies

For peer review only - http://bmjopen.bmj.com/site/about/guidelines.xhtml

reporting increased rates of all common mental health conditions in children[7]. Moreover, fatigue has also been studied in paediatric long-term conditions including CYP with multiple sclerosis[8 9]. Emerging clinical observations and preliminary research indicate that COVID-19 can have a long-term impact on CYP in a range of domains[10 11] but well conducted, methodologically robust studies are lacking.

This study primarily aims to (a) describe the clinical phenotype and prevalence of post-COVID symptoms (e.g., pain or physical symptoms, fatigue, sleep problems, mental health problems) among test positive and test negative CYP, (b) produce an operational definition of Long COVID in CYP, and (c) establish the prevalence of Long COVID in CYP testing positive for SARS-CoV-2 infection. The second aim is of particular importance given that this is a prerequisite for any future epidemiological or interventional study. This will be achieved by using the empirical data we will acquire from 17,000 CYP and a national Delphi consensus process.

Methods

Study design and setting

Public Health England (PHE) has been conducting national surveillance of SARS-CoV-2 since the start of the pandemic in England. PHE receives daily electronic notifications of all SARS-CoV-2 RT-PCR tests performed in healthcare settings (Pillar 1 tests) and in the community (Pillar 2 tests), which are reported through the Second Generation Surveillance System (SGSS). Information within the SGSS reports includes NHS number, name, age, sex, postcode, date of sample, reporting laboratory and test result. PHE also has access to the electronic Patient Demographic Service (PDS), which contains the names, addresses and status (alive/dead) of all patients registered with the NHS.

A sample of CYP aged 11-17 years old when testing positive for SARS-CoV-2 between September 2020 and March 2021 and COVID test-negative CYP matched for age, sex and region identified through SGSS will be linked to PDS using available identifiers and postal addresses. A letter will be posted to them, informing them about the study and inviting them to take part using an online link. This link will provide them with information about the study, with an option to consent online and complete a short recruitment questionnaire (or both as paper options). Recruitment was planned to start in March 2021 and all data collection anticipated to end in April 2023.

This will be a cohort study of SARS-CoV-2 test-positive CYP aged 11-17 years matched on the test date, age, sex and geography to SARS-CoV-2 test-negative controls identified by PHE.

Participants

Between September 2020 and March 2021, 234,803 CYP aged between 11 and 17 years were diagnosed with COVID-19 in England. During the same period there were 1,481,154 negatives tests among this age group equivalent to 1,203,996 CYP. Among those who tested negative

For peer review only - http://bmjopen.bmj.com/site/about/guidelines.xhtml

76,689 individuals (100,154 tests) were excluded as they had a positive result pre and/or post their negative test. The positive and negative cohorts were linked using age at test, gender, month of test, and lower super output area resulting in 102,402 positive individuals and 147,561 negative individuals. Matched individuals were checked against the NHS spine to exclude any individuals who have died, and to extract participants addresses to send invitation letters. 37 individuals were excluded because they had died since their COVID-19 test (6 among those positive and 31 among those negative), and 11,193 individuals of the positive cohort and 19,251 individuals of the negative cohort were excluded as an address was not available. Finally, 246 CYP were excluded as they were included in a previous study. Following exclusions 91,016 positive CYP and 128,220 negative CYP will be available to be invited to participate in the study. For all the months except December, all eligible individuals will be sent an invitation. For those tested in December, 2,970 positive CYP and 5,911 negative CYP will be invited as the eligible number was so large that additional funding would be needed to invite all those eligible.

This is shown schematically in Figure 1.

Data collection

Participating families of CYP will be contacted 3, 6, 12 and 24 months after the CYP's SARS-CoV-2 test (depending on recruitment month) and invited to take part in the study. Following online informed consent, the CYP will self-complete an online (or paper) questionnaire about their mental and physical health. CYP towards the lower end of the 11-17 age band and CYP with special educational needs or disability may require the help of a carer to complete the questionnaire.

Outcomes

The questionnaires given to CYP include elements of the ISARIC Paediatric COVID-19 follow-up questionnaire[12] and the recent Mental Health of Children and Young people in England surveys[13] to facilitate international comparisons regarding the risk factors and profile of Long COVID in CYP. The questionnaires given to CYP were designed together with the International Severe Acute Respiratory and emerging Infection Consortium (ISARIC) Paediatric Working Group to produce a harmonised data collection tool, allowing for standardisation and metanalysis by design.

The elements taken from the ISARIC Paediatric COVID-19 follow-up questionnaire[12] include questions about demographics and physical symptoms, with particular questions enquiring about cough and fever, given that these are the main acute symptoms in non-hospitalised CYP[14] and gastrointestinal symptoms, as a study has shown such symptoms were common in seropositive CYP[15]. It is likely other symptoms will manifest later in Long COVID (e.g., tiredness, headaches, myalgia etc.), with the skin rashes and cardiac problems in hospitalised children with paediatric multisystem inflammatory syndrome being borne in mind for Long COVID[5]. Those older CYP could very well overlap with adult symptomatology (e.g., a neuropsychiatric-encephalitic sub-group).

For peer review only - http://bmjopen.bmj.com/site/about/guidelines.xhtml

In terms of the other questions, the Strengths and Difficulties Questionnaire (SDQ)[16] will be used to assess emotional and mental health, consisting of 25 items. The EQ-5D-Y[17] will be used as a measure of quality of life/functioning, with fatigue measured using the 11-item Chalder Fatigue Questionnaire (CFQ)[18]. Additionally, the short, 7-item version of The Warwick Edinburgh Mental Wellbeing Scale (WEMWBS)[19] will be included. Loneliness will be measured using the 4-item UCLA Loneliness Scale for Children[20] as well as self-reported school attendance.

The length of the online self-assessment has an estimated completion time of approximately 20 minutes for CYP. The CYP do not have to complete all of the questions at once but can pause and save the answers at any point.

Statistical analyses

Response rates for positive and negative CYP will inform expectations of final study size and possibly refinement of the sampling percentages. Descriptive statistics will be used for all dimensions collected at baseline and the follow-up questionnaires, separately by test status, wave and month of test. Appropriate data visualisation techniques will be used to examine trajectories over time for those variables that are measured repeatedly, separately by test status and month of test. Summary measures of the fatigue and mental health dimensions will be generated using dimension reduction methods such as latent variables/latent class/PCA methods. Cross-tabulation of latent variables against test status as well as discriminant analysis will help operationalise preliminary definitions of Long COVID. These will be compared with definitions based on categorisation of prolonged symptoms supplemented by a Delphi consensus process.

Sample size

A total of 5,000 participants (2,500 test-positive and 2,500 test-negative) would give 80% power to detect at least a 4% difference in symptoms at 5% significance, if the COVID-19 negative participants had a prevalence similar to that reported for adults (i.e., 34%). Greater baseline prevalences (e.g., 40%) would require slightly greater numbers for the same power and confidence; smaller baseline prevalences (e.g., 20%) smaller numbers. However, identifying risk factors for long COVID-19 would require studying demographic, social, family and clinical data and thus we would require larger numbers. For this reason, we are planning to invite all available participants.

Patient and Public Involvement

Resources have been allocated for any Patient and Public Involvement (PPI) activities at INVOLVE rates including a nationally representative PPI research advisory group. PPI members will be offered training and support in accordance with the INVOLVE[21] report and other guidance[22] including online training. PPI meetings will take place quarterly.

For peer review only - http://bmjopen.bmj.com/site/about/guidelines.xhtml

Ethics and Dissemination

Ethical considerations

PHE has legal permission, provided by Regulation 3 of The Health Service (Control of Patient Information) Regulations 2002, to process patient confidential information for national surveillance of communicable diseases. Individual patient consent is not required for initial invitation to the study. Parents/carers and young people will be sent an invitation with a link to the website with the relevant information sheets and consent forms. They will have the opportunity to ask any questions about the study. Parents/carers of CYP under 16 years of age will be asked to complete a Parent/carer consent form. The young person will also be asked to sign an assent form to indicate their agreement. 16-17-year-olds will be asked for consent (using the Young Person Consent Form); but their parents will not. This is in line with Health Research Authority recommended processes[23].

Study approved by Yorkshire & The Humber - South Yorkshire Research Ethics Committee (REC reference: 21/YH/0060; IRAS project ID:293495).

It is possible that the questionnaires may make some vulnerable participants feel fatigued or distressed from completing questionnaires relating to their mental health and/or report serious symptoms that put them at immediate risk. The research team will provide information on where to seek support and provide self-help information. Unfortunately, the researchers are unable to provide medical advice. However, existing national surveys of children's mental health also follow this risk protocol.

Dissemination

Peer reviewed publications, briefings for policy makers and lay summaries for participants and CYP and carers. Results will be made public, initially on a pre-print server and we will allow reuse of articles under a CC BY licence. The dataset will be made publicly available.

For peer review only - http://bmjopen.bmj.com/site/about/guidelines.xhtml

Figure 1: Flowchart of the selection process.

For peer review only

For peer review only - http://bmjopen.bmj.com/site/about/guidelines.xhtml

Author contributions:
Terence Stephenson t.stephenson@ucl.ac.uk conceived the idea for the study, submitted the successful grant application and undertook the first revision of the manuscript.
Roz Shafran r.shafran@ucl.ac.uk provided ideas on mental health follow up to the original grant application, submitted the ethics and R&D applications and revised the manuscript.
Bianca De Stavola b.destavola@ucl.ac.uk provided statistical input to the design and analysis, including sample size calculations.
Natalia Rojas n.rojas@ucl.ac.uk wrote the first draft of the manuscript based on the grant application, protocol and ethics form and compiled the references.
Felicity Aiano Felicity.Aiano@phe.gov.uk helped develop the study design
Zahin Amin-Chowdhury Zahin.Amin@phe.gov.uk helped develop the study design
Kelsey McOwat Kelsey.Mcowat@phe.gov.uk adapted the questionnaire for the online SNAP survey platform.
Maria Zavala Maria.Zavala@phe.gov.uk undertook a separate pilot study which informed the CLoCk consent process and the online questionnaire.
Ruth Simmons Ruth.Simmons@phe.gov.uk designed the participant sampling and dataflow.
Shamez Ladhani shamez.ladhani@phe.gov.uk operationalised the regulatory and recruitment ideas for the study and revised the manuscript.

Olivia Swann and Elizabeth Whittaker designed the elements of the ISARIC Paediatric COVID-19 follow-up questionnaire which were incorporated into the online questionnaire used in this study to which all the CLoCk Consortium members contributed.

All members of the CLoCk Consortium made substantial contributions to the conception or design of the work; were involved in drafting both the funding application and this manuscript; approved the version to be published; and agree to be accountable for all aspects of the work in ensuring that questions related to the accuracy or integrity of any part of the work are appropriately investigated and resolved.

Co-Applicants on the grant application and CLoCk Consortium members (alphabetical):
Marta Buszewicz, University College London
Trudie Chalder, King's College London, trudie.chalder@kcl.ac.uk
Esther Crawley, University of Bristol, Esther.Crawley@bristol.ac.uk
Emma Dalrymple, PPI representative, emmajdalrymple@gmail.com
Tamsin Jane Ford, University of Cambridge, tjf52@medschl.cam.ac.uk
Shruti Garg, University of Manchester, Shruti.Garg@mft.nhs.uk
Anthony Harnden, University of Oxford, anthony.harnden@phc.ox.ac.uk
Dougal Hargreaves, Imperial College London, Dougal.HARGREAVES@education.gov.uk
Isobel Heyman, Great Ormond Street Hospital for Children NHS Foundation Trust, i.heyman@ucl.ac.uk,
Michael Levin, Imperial College London, m.levin@imperial.ac.uk
Vanessa Poustie, University of Liverpool, v.poustie@liverpool.ac.uk
Terry Segal, University College London Hospitals NHS Foundation Trust, terry.segal@nhs.net
Malcolm Semple, University of Liverpool, M.G.Semple@liverpool.ac.uk

For peer review only - http://bmjopen.bmj.com/site/about/guidelines.xhtml

Kishan Sharma, Manchester University NHS Foundation Trust, Kishan.Sharma@mft.nhs.uk
Olivia Swann, University of Edinburgh, olivia.swann@ed.ac.uk
Elizabeth Whittaker, Imperial College London, e.whittaker@imperial.ac.uk

Acknowledgement: Michael Lattimore, Public Health England, as Project Officer for the CLoCk study

Competing interests statement: Terence Stephenson is Chair of the Health Research Authority and therefore recused himself from the Research Ethics Application

Funding statement: Funded by The Department of Health and Social Care, in their capacity as the National Institute for Health Research (NIHR), and by UK Research & Innovation (UKRI) who have awarded funding grant number COVLT0022. All research at Great Ormond Street Hospital NHS Foundation Trust and UCL Great Ormond Street Institute of Child Health is made possible by the NIHR Great Ormond Street Hospital Biomedical Research Centre. The views expressed are those of the author(s) and not necessarily those of the NHS, the NIHR or the Department of Health

Data Sharing: Applications for relevant anonymised data should be submitted to the PHE office for Data Release: https://www.gov.uk/government/publications/accessing-public-health-england-data/about-the-phe-odr-and-accessing-data

For peer review only - http://bmjopen.bmj.com/site/about/guidelines.xhtml

References

1. Orben A, Tomova L, Blakemore S-J. The effects of social deprivation on adolescent development and mental health. *The Lancet Child & Adolescent Health* 2020;4(8):634-40. doi: 10.1016/s2352-4642(20)30186-3
2. Whittaker E, Bamford A, Kenny J, et al. Clinical Characteristics of 58 Children With a Pediatric Inflammatory Multisystem Syndrome Temporally Associated With SARS-CoV-2. *JAMA* 2020;324(3):259-69. doi: 10.1001/jama.2020.10369 [published Online First: 2020/06/09]
3. Swann OV, Holden KA, Turtle L, et al. Clinical characteristics of children and young people admitted to hospital with covid-19 in United Kingdom: prospective multicentre observational cohort study. *BMJ* 2020;370:m3249. doi: 10.1136/bmj.m3249 [published Online First: 2020/09/23]
4. Pierce M, Hope H, Ford T, et al. Mental health before and during the COVID-19 pandemic: a longitudinal probability sample survey of the UK population. *The Lancet Psychiatry* 2020;7(10):883-92. doi: 10.1016/s2215-0366(20)30308-4
5. Ludvigsson JF. Case report and systematic review suggest that children may experience similar long-term effects to adults after clinical COVID-19. *Acta Paediatr* 2021;110(3):914-21. doi: 10.1111/apa.15673 [published Online First: 2020/11/19]
6. Sudre CH, Murray B, Varsavsky T, et al. Attributes and predictors of Long-COVID: analysis of COVID cases and their symptoms collected by the Covid Symptoms Study App. 2020 doi: 10.1101/2020.10.19.20214494
7. Moore DA, Nunns M, Shaw L, et al. Interventions to improve the mental health of children and young people with long-term physical conditions: linked evidence syntheses. *Health Technol Assess* 2019;23(22):1-164. doi: 10.3310/hta23220 [published Online First: 2019/05/28]
8. Carroll S, Chalder T, Hemingway C, et al. "It feels like wearing a giant sandbag." Adolescent and parent perceptions of fatigue in paediatric multiple sclerosis. *Eur J Paediatr Neurol* 2016;20(6):938-45. doi: 10.1016/j.ejpn.2016.06.004 [published Online First: 2016/07/17]
9. Carroll S, Chalder T, Hemingway C, et al. Understanding fatigue in paediatric multiple sclerosis: a systematic review of clinical and psychosocial factors. *Dev Med Child Neurol* 2016;58(3):229-39. doi: 10.1111/dmcn.12964 [published Online First: 2015/11/15]
10. Buonsenso D, Espuny Pujol F, Munblit D, et al. Clinical Characteristics, Activity Levels and Mental Health Problems in Children with Long COVID: A Survey of 510 Children. *Preprints* 2021 doi: 10.20944/preprints202103.0271.v1
11. Munblit D, De Rose C, Sinatti D, et al. Preliminary Evidence on Long COVID in children. *medRxiv* 2021
12. Sigfrid L, Maskell K, Bannister PG, et al. Addressing challenges for clinical research responses to emerging epidemics and pandemics: a scoping review. *BMC Med* 2020;18(1):190. doi: 10.1186/s12916-020-01624-8 [published Online First: 2020/06/27]
13. NHS Digital. Mental Health of Children and Young People in England, 2020: Wave 1 follow up to the 2017 survey 2020 [Available from: https://tinyurl.com/NHSCYPFU17 accessed 13 April 2021.
14. Ladhani SN, Amin-Chowdhury Z, Davies HG, et al. COVID-19 in children: analysis of the first pandemic peak in England. *Arch Dis Child* 2020;105(12):1180-85. doi: 10.1136/archdischild-2020-320042 [published Online First: 2020/08/17]
15. Waterfield T, Watson C, Moore R, et al. Seroprevalence of SARS-CoV-2 antibodies in children: a prospective multicentre cohort study. *Arch Dis Child* 2020 doi: 10.1136/archdischild-2020-320558 [published Online First: 2020/11/12]

For peer review only - http://bmjopen.bmj.com/site/about/guidelines.xhtml

16. Goodman R. Psychometric properties of the strengths and difficulties questionnaire. *J Am Acad Child Adolesc Psychiatry* 2001;40(11):1337-45. doi: 10.1097/00004583-200111000-00015 [published Online First: 2001/11/09]
17. Wille N, Badia X, Bonsel G, et al. Development of the EQ-5D-Y: a child-friendly version of the EQ-5D. *Qual Life Res* 2010;19(6):875-86. doi: 10.1007/s11136-010-9648-y [published Online First: 2010/04/21]
18. Chalder T, Berelowitz G, Pawlikowska T, et al. Development of a fatigue scale. *J Psychosom Res* 1993;37(2):147-53. doi: 10.1016/0022-3999(93)90081-p [published Online First: 1993/01/01]
19. Tennant R, Hiller L, Fishwick R, et al. The Warwick-Edinburgh Mental Well-being Scale (WEMWBS): development and UK validation. *Health Qual Life Outcomes* 2007;5:63. doi: 10.1186/1477-7525-5-63 [published Online First: 2007/11/29]
20. Office of National Statistics. Children's and young people's experiences of loneliness: 2018 2018 [Available from: https://tinyurl.com/CYPExperiencesOfLoneliness accessed 13 April 2021.
21. INVOLVE. Developing training and support for public involvement in research 2012 [Available from: https://www.invo.org.uk/wp-content/uploads/2012/11/INVOLVETrainingSupport2012.pdf accessed 13 April 2020.
22. National Institute for Health Research (NIHR). PPI (Patient and Public Involvement) resources for applicants to NIHR research programmes. 2019 [Available from: https://tinyurl.com/PPIApplicantResources accessed 13 April 2021.
23. Health Research Authority (HRA). Research involving children 2018 [Available from: https://www.hra.nhs.uk/planning-and-improving-research/policies-standards-legislation/research-involving-children/ accessed 13 April 2021.

For peer review only

For peer review only - http://bmjopen.bmj.com/site/about/guidelines.xhtml

Figure 1: Flowchart of the selection process.

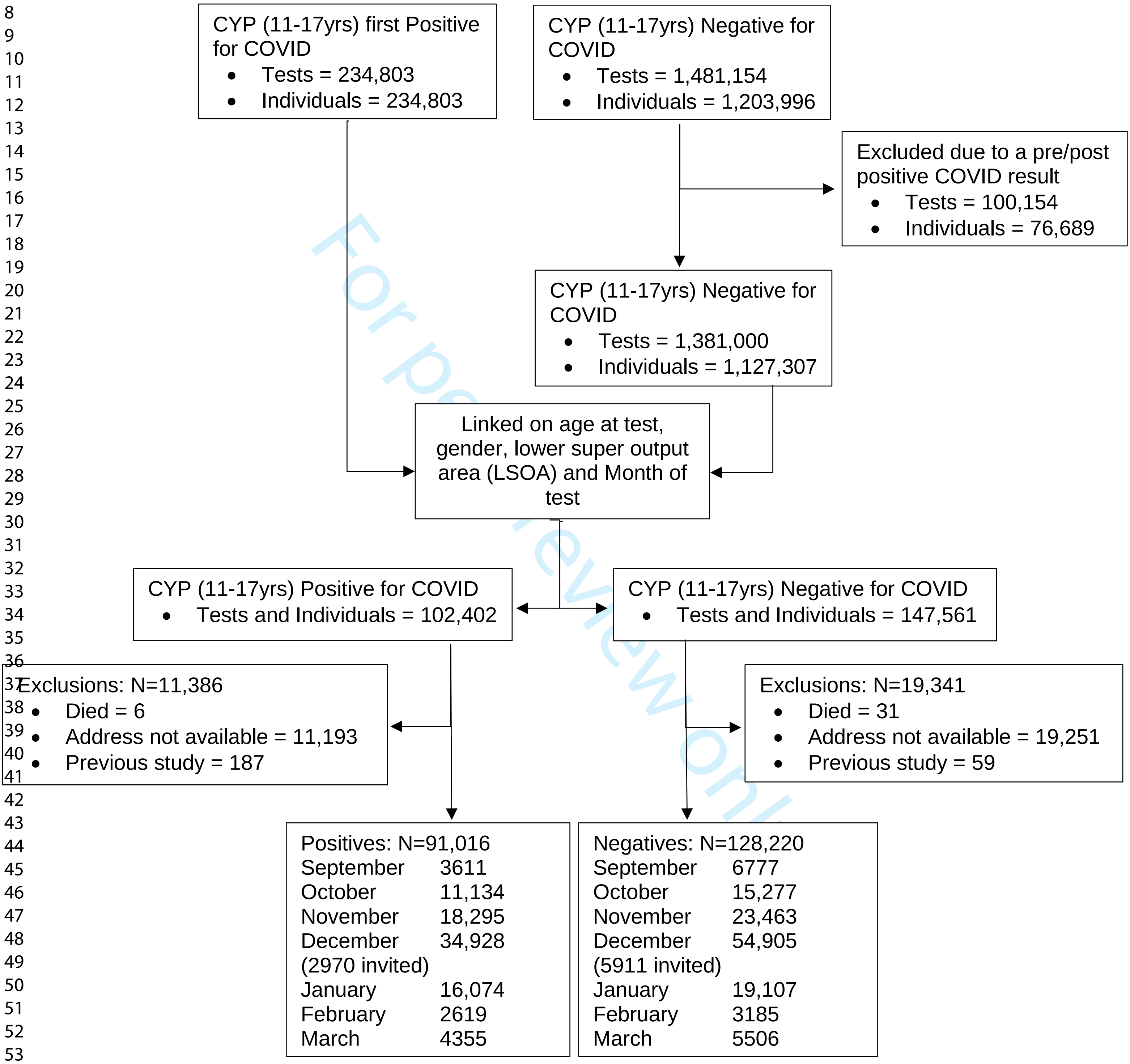

For peer review only
