# Supplementary material for: Long COVID and the mental and physical health of children and young people: national matched cohort study protocol (the CLoCk study)
Source: BMJ Open. 2021 Aug 26;11(8):e052838. doi: 10.1136/bmjopen-2021-052838 (PMC8392739; doi:10.1136/bmjopen-2021-052838)
Supplement: Reviewer comments [file bmjopen-2021-052838.reviewer_comments.pdf]

## PEER REVIEW HISTORY

BMJ Open publishes all reviews undertaken for accepted manuscripts. Reviewers are asked to complete a checklist review form (**http://bmjopen.bmj.com/site/about/resources/checklist.pdf**) and are provided with free text boxes to elaborate on their assessment. These free text comments are reproduced below.

## ARTICLE DETAILS

| | |
|---|---|
| **TITLE (PROVISIONAL)** | Long Covid and the mental and physical health of children and young people: national matched cohort (CLoCk) study protocol |
| **AUTHORS** | Stephenson, Terence; Shafran, Roz; De Stavola, Bianca; Rojas, Natalia; Aiano, Felicity; Amin-Chowdhury, Zahin; McOwat, Kelsey; Simmons, Ruth; Zavala, Maria; CLoCk, Consortium; Ladhani, Shamez N. |

This article was not externally reviewed at BMJ Open. Protocols that have been independently assessed prior to submission to BMJ Open are usually fast-tracked to publication on the grounds that further substantial changes will not be possible. This independent assessment will usually be external, independent review for both a substantial grant award from a non-commercial or government funder and ethics approval.
